# How important is the spatial movement of people in attempts to eliminate the transmission of human helminth infections by mass drug administration?

**DOI:** 10.1098/rstb.2022.0273

**Published:** 2023-10-09

**Authors:** Benjamin S. Collyer, James E. Truscott, Charles S. Mwandawiro, Sammy M. Njenga, Roy M. Anderson

**Affiliations:** ^1^ Department of Infectious Disease Epidemiology, School of Public Health, Faculty of Medicine, St Mary's Campus, Imperial College London, London W2 1PG, UK; ^2^ Kenya Medical Research Institute, Nairobi, Kenya; ^3^ Eastern and Southern Africa Centre of International Parasite Control, Kenya Medical Research Institute, Nairobi, Kenya

**Keywords:** soil-transmitted helminths, spatial transmission model, hookworm

## Abstract

Human mobility contributes to the spatial dynamics of many infectious diseases, and understanding these dynamics helps us to determine the most effective ways to intervene and plan surveillance. In this paper, we describe a novel transmission model for the spatial dynamics of hookworm, a parasitic worm which is a common infection across sub-Saharan Africa, East Asia and the Pacific islands. We fit our model, with and without mobility, to data obtained from a sub-county in Kenya, and validate the model's predictions against the decline in prevalence observed over the course of a clustered randomized control trial evaluating methods of administering mass chemotherapy. We find that our model which incorporates human mobility is able to reproduce the observed patterns in decline of prevalence during the TUMIKIA trial, and additionally, that the widespread bounce-back of infection may be possible over many years, depending on the rates of people movement between villages. The results have important implications for the design of mass chemotherapy programmes for the elimination of human helminth transmission.

This article is part of the theme issue ‘Challenges and opportunities in the fight against neglected tropical diseases: a decade from the London Declaration on NTDs’.

## 1. Introduction

The measurement and prediction of the spatial distribution and spread of infection within and between defined population units, e.g. towns, cities and villages, are vital considerations for planning epidemiological interventions [1–3]. This is in stark focus today, as the globe confronts the global COVID-19 pandemic which is causing millions of cases of severe illness, concomitant high levels of mortality and unprecedented disruption of daily life in modern times. The spatial transmission of infection via people movements within and between towns, countries and larger areas are vital to an understanding of how best to control the spread of SARS-CoV-2 by contact tracing, social distancing measures and mass vaccination.

Diseases caused by helminth worm infections, affect over two billion people worldwide and are endemic in many of the world's low- and middle-income countries [4,5]. Large-scale trials, where neglected tropical disease infections are mapped and the effectiveness of interventions are measured, are becoming increasingly common [6–9]. However, the development of mechanistic models which can accurately capture the spatial transmission of helminths, created either by the movement of people or intermediate hosts and vectors, and make predictions over large regions, has so far lagged behind the acquisition of spatial data, and its statistical analysis [10]. Much of the analyses of spatial patterns of infection are descriptive in nature and do not address the underlying dynamics of spatial transmission [11,12]. The spatial dynamics, as opposed to spatial description of patterns of infection, is very important in the design of both measurement units in the monitoring and evaluation of control programmes and in the design of the control programme itself. Currently, there are no existing models for diseases caused by soil-transmitted helminths, to our knowledge, that explicitly take space and people movements into consideration within a dynamic framework, and most are applicable only to small populations. This is in marked contrast to other areas of infectious disease epidemiology [13].

Space is an important factor to take into consideration for understanding patterns of both directly and indirectly spread infectious agents that are endemic in large populations, because while normally the majority of transmission is local, movement of humans (or disease vectors) may affect transmission elsewhere. This is especially the case when prevalence is focal, as it is often uncertain when ongoing transmission is sustained purely locally or only because of influence from further away by the repeated input of new infections from other spatial locations. In such situations, it is useful to know how efforts to control the disease in one location affects the dynamics of infection in other surrounding areas or if infection is likely to return after control has ended with the elimination of transmission in a defined area. Many studies, reveal much heterogeneity in the spatial pattern of helminth infection prevalence across multi-village communities [8,9,14,15].

When human movement is completely random, diffusion models can be used to model spatial spread [16,17]. However, observed patterns of human movement, independent of country, follow more predictable patterns influenced by highways of movement between and within centres of population. Day-to-day movements typically take the form of repeated short distance moves between place of residence and, for example, place of occupation, education, retail and leisure. Longer distance commuter patterns between high-density populated areas occur more frequently than pure diffusion would allow [18]. Modelling approaches to capture realistic patterns of the dynamics of spatial movement on patterns of infection include metapopulation models, network and lattice-based models and partial-integro-differential equations. These types of approaches have been applied to better understand the spread and control of foot-and-mouth disease, measles and pandemic influenza [19–23].

### (a) Spatial models of helminth transmission and control

Human helminth infections enter a human host as larval stages, reach maturity within the human host and reproduce sexually to release further transmission stages to infect either an intermediate host such as an insect or snail or a further human host. Their offspring leave the body as fertilized eggs or larval stages, and transmission between people occurs indirectly, either through environmental reservoirs of contaminated soil, water or via vector species [4].

Models of helminth transmission differ from models of micro-parasites because the generation time of helminth species are far longer (typically between 1 and 10 years depending on the species) than that of bacteria and viruses, and the infectiousness of an infected individual is strongly dependent on their burden [24]. Because of these differences, it is vital to account for the heterogeneity of burdens within the locality under consideration. Heterogeneity of burden in the population is commonly observed in empirical surveys of infection, where it is typical to observe that the majority of individuals in localized surveys of endemic areas harbour small burdens, and a small number of individuals harbour very large burdens and are presumed to contribute disproportionally to ongoing transmission. The negative binomial probability distribution provides a good empirical description of worm burdens per human host [24]. Sources of the heterogeneity within small localities are generally assumed to be a product of unequal risk (owing to occupation, access to adequate sanitation, housing quality, etc.) and predisposition (perhaps triggered by acquired immunity, or genetic background).

One of the earliest models for helminth transmission was proposed by Anderson & May where the heterogeneity in burden is described by assuming worm burdens per person in a closed localized population have a negative binomial distribution [25–27]. They derived ordinary differential equations describing how the mean of this distribution, and density of the infectious material in a reservoir, evolves over time and under the impact of control interventions. These models showed clearly that in the absence of any acquired immunity generated by repeated infection with helminth parasites, once mass drug treatment ceased, parasite populations would bounce back to pre-control levels unless treatment reduced prevalence below a transmission breakpoint. Extensions to account for various complications such as age structure, acquired immunity, mating structure of species, density-dependent egg production, variation in host genetic background and the dynamics of vector populations have also been developed. More recently, a trend has developed towards the development of individual-based stochastic models where each person's worm burden in a defined host population is a discrete entity [28–31]. These models lose analytic tractability, but are able to capture specific details such as host demography, individual variation in exposure to infection and interventions targeted at certain sections of the human host population such as specific age groups.

The population size for which such models are appropriate for, is dependent on how many people live within an area in which parasite transmission can be considered local. What constitutes ‘local’ for helminth transmission depends on the route of the exposure to infection. For example, if the route is contaminated water, as is the case for schistosome transmission, the locality is determined by the residing locations of people who use and concomitantly contaminate the source; or, for example, if the helminth is transmitted by an insect vector (e.g. lymphatic filariasis and onchocerciasis), the locality is defined by the range and habitat of the insect. To date, the majority of models of helminth transmission have focused on applications where all transmission is local or where non-local transmission is accounted for by assuming a constant rate of infectious material entering an infectious reservoir. This has been appropriate because generally the models are applied to transmission in villages in rural settings where it can be reasonably assumed the majority of cases arise from local transmission within a village.

Increasingly, attention is being focused on transmission in settings where previously endemic areas have received intensive deworming through repeated rounds of mass chemotherapy. The past two decades have seen great progress in many countries with endemic helminth infections in reducing the prevalence of infection as a result of preventative chemotherapy targeted at either school-aged children in the case of soil-transmitted helminths and schistosome parasites, or the whole community in the case of the vector-transmitted filarial worms. As prevalence falls year by year, marked heterogeneity in infection levels is often observed owing to local differences in both the coverage and compliance to treatment, and environmental conditions. In these circumstances, it is be important to develop an understanding of what influences the chance of elimination of transmission in the long term in a defined locality, and what are the chances of infection resurgence post mass drug administration (MDA) cessation. Within these settings, owing to the patchy spatial pattern of prevalence left after deworming, it is possible that non-local transmission may become the dominating factor influencing the re-emergence of widescale transmission. Therefore, models that account for people movements linked to spatial parasite transmission over large scales are needed. The ground-breaking recent work of Touloupou and Retkute, which links geostatistical mapping, a helminth transmission model and a statistical importance sampling method to produce very large-scale predictive maps, does include an element of spatial transmission, although does not incorporate observed people movement patterns [32,33].

In this paper, we create an explicitly spatial model for hookworm transmission, which incorporates realistic human movement patterns that facilitate the spatial spread of infection. Our spatial model is designed to mimic the transmission of soil-transmitted helminths with a focus on hookworm. However, it is easily adapted for other human helminth infections. We aim to address a number of questions. The first is: can our model predict the decline in hookworm prevalence observed in the TUMIKIA study, over the course of repeated rounds of MDA [9]. The second is: does it perform better than a model that does not account for observed people movement patterns between villages? The third and perhaps most important question is: how does the inclusion of human movement affect the chances of transmission elimination over a spatial scale involving many villages as described in the TUMIKIA study? To investigate this question, we will simulate a high coverage MDA treatment programme and determine the chance of local elimination events, with and without human movements between villages.

## 2. Methods

We have created a meta-population hookworm transmission model, consisting of subpopulations modelled by an individual-base stochastic framework in which human hosts are able to temporarily make visits of varying durations to other subpopulations. The area under consideration is discretized onto a regular grid, and each cell—or pixel—contains a subpopulation consisting of permanent residents of the pixel who share access to a reservoir of infectious material. The dimensions of the pixels in this study are 3 km x 3 km, and in general should be chosen so that the population shares some access to the same collective reservoir of infective stages within normal day-to-day movements.

### (a) Subpopulation model structure

Each subpopulation consists of an individual-based model, similar to those employed previously in a number of studies which are based upon a stochastic version of Anderson and May's original helminth transmission model [29,31,34]. Human hosts can become infected with hookworm when they are in direct contact with soil that is contaminated by the infective larval hookworm stage. The larvae are able to penetrate the skin, typically through the feet and travel through the body to the small intestine where they mature into adult worms. The worms reproduce sexually inside the small intestine, where the female produces valid eggs, which are passed out in the host's faeces. If the eggs are deposited in warm, moist soil then the hatched eggs are able to develop into infective larval stages.

In our model, mature male and female worms within an individual are denoted and $W_{f}^{i}$, respectively (where *i* indexes the person). To generate over-dispersed worm burdens, each individual host has a parameter, *c*, generated from a Gamma distribution. This variable represents the individual's predisposition to infection relative to other individuals in the subpopulation, which can be considered to be the product of genetic, social, behavioural or environmental factors. Typically, exposure to infection is expected to be dependent on age, however prior analysis of the TUMIKIA hookworm dataset found no evidence of age dependence, so age dependence is not included in this analysis. We model the uptake of worms in an individual as a Poisson process with the rate given by product of the pixel's mean contact rate, the individual's predisposition, and the concentration of larvae in the reservoir. We simulate the Poisson process using the τ-leap method. Mature adult worms mate polygamously (one male can mate with multiple females), and the production of fertilized eggs, which decays exponentially with female worm burden, are output into the environmental reservoir. The mean concentration of infective larval stages in the local reservoir is denoted *L*. The details of the transmission process are given in table 1.

**Table 1.** Event table for the stochastic individual-based model of hookworm transmission. (*i* indexes individual hosts in a single subpopulation.)

event		rate
infection	$I_{i}∼\mathrm{Poisson}(\beta c_{i}L\Delta t)$ (new infections)	each timestep
	$f_{i}∼\mathrm{binomial}(I_{i},0.5)$ (new females)	
	$W_{m}^{i}\to W_{m}^{i}+I_{i}-f_{i}$	
	$W_{f}^{i}\to W_{f}^{i}+f_{i}$	
mature worm death	$W_{m}^{i}\to W_{m}^{i}-1$	$1/\delta_{w}$
	$W_{f}^{i}\to W_{f}^{i}-1$	
egg production	$E_{i}=\alpha \;W_{f}^{i}\;(W_{m}^{i}>0)\exp(-\gamma W_{f}^{i})$	each timestep
faecal sample	$EPG_{i}∼\mathrm{negbin}(E_{i},k_{e})$	each timestep
reservoir gain	$L\to L+\underset{i}{\sum }E_{i}$	each timestep
reservoir loss	$L\to \left(\frac{1-\delta_{e}}{\Delta t}\right)L$	each timestep
human death/birth	$W_{m}^{i}=0,W_{f}^{i}=0$,	$\frac{1}{\delta_{h}}$
	$c_{i}\tilde{\mathrm{gamma}}\left(\frac{1,1}{k}\right)$	

### (b) Trips

In our metapopulation model, the spatial spread of hookworm infection is facilitated by visits taken by individuals to locations in other pixels, We assume that the majority of travel an individual makes of duration less than a day, for example, school attendance or commuting for work, will be within their home pixel and is modelled by within cell transmission dynamics, therefore visits outside the home cell are assumed to last longer than a day, While on a visit, an individual is assumed to be exposed to infectious contact with larval stages in that environment, and are able to contribute to, and be infected by, the local reservoir of infective stages.

For simplicity, we have each individual in the population equally likely to make visits (in reality, the propensity to take trips is strongly dependent on age, and other sociological factors which may, in some individuals, be correlated with worm burden). In each discrete timestep of the individual-based model, every individual in cell *i* has a probability of taking a trip to cell *j*, which we denote as $p_{ij}$. We estimate $p_{ij}$ using the gravity model developed by Marshall *et al*. [35], originally for malaria transmission, in which the rate of individuals taking trips to other cells is dependent on the population size in the source and destination pixel, in addition to the distance between them:

$$p_{ij}=\frac{\theta N_{j}^{\tau }}{{\left(1+\rho \;d_{ij}\right)}^{\sigma }}.$$

In the equation above, $N_{j}$ represents the population at destination *j*, $d_{ij}$ is the Euclidean distance between two locations and  τ, ρ and σ are parameters estimated from data and dependent on the area being modelled. θ is the normalization constant. In this study, we estimate this parameter, which factors in the relative chance that a person on a trip has of being exposed to infectious material compared to a local resident.

Each visit has a duration, that is, the length of time spent away from the resident pixel in a single visit, which is also generated randomly from a predefined probability distribution, where shorter duration trips are more likely than longer ones. Our model uses a fixed timestep of one month, therefore, the fractional part of the visit duration in months is used as a multiplicative factor of that individual's exposure in that month. We use a previously published empirical distribution from Namibia taken from call data records which recorded 259 million trips by 2.5 million mobile phone subscribers between 2010 and 2014 [36] (table 2).

**Table 2.** Empirical cumulative distribution of trip lengths [36].

duration, *d* (days)	probability of trip length ≤*d*
1	0.47
3	0.71
7	0.85
14	0.92
30	0.96
60	0.98
90	0.99

The gravity model effectively generates a complete graph of interactions between subpopulations, in which there is a non-zero probability of individuals taking trips to all other pixels. To aid computational efficiency, we employ a minimum threshold set so that the expected number of trips between each pair of locations is no less than once per year, to filter out low probability trips. This supresses the possibility of low probability events, and may influence the tail of sampled distributions (in some circumstances changing the mean dynamics of transmission). However, we believe it will have limited bearing on overall results.

### (c) Data sources

The TUMIKIA trial was conducted between 2015 and 2017 in Kwale sub-county, Kenya, where three helminth species are endemic with hookworm being the most prevalent infection [9,37,38]. The clustered randomized control trial evaluated the efficacy of annual and binannual community-based deworming against the routine standard of care, provided by annual school-based treatment. The trial split locations into three arms, annual community-wide treatment (arm A), biannual community-wide treatment (arm B) and school-based treatment (arm C). Deworming was achieved by use of the drug albendazole. Prevalence data for each arm of the study were collected at three time points, baseline (before treatment for hookworm), and 12 and 24 months after the first intervention. The midline survey was conducted four months after round 2, and two months prior to round 3. The endline survey was conducted two months after round 4. In each community, 255 households were randomly selected to participate in the survey, with one household member randomly asked to provide a stool sample. Overall treatment coverage in the community-wide treatment group ranged from 74 to 82% across the four rounds, while coverage in school-age children was consistently high at 88% in both years. The baseline survey included 19 684 individuals, the 12-month assessment survey included 24 843 individuals and the endline survey included 22 188 individuals.

The recorded location and hookworm positivity status of each participant were aggregated to obtain the number of samples and number of positive cases in each pixel of a 3 km x 3 km grid. The resolution was chosen to roughly approximate the area of a community. To create baseline, midline and endline maps with estimates of the prevalence in each pixel, we fitted binomial logistic models to the data using a Monte Carlo maximum-likelihood method, using the PrevMap R package [11]. The model assumes that the random effects in the logit transform of the prevalence data are the sum of a zero-mean Gaussian process with Matern correlation function, and an independent zero-mean Gaussian random variable.

Population size in each pixel was obtained from estimates produced by Facebook Data for Good, downloaded from the Humanitarian Data Exchange [39]. The total estimated population in the whole area was 738 027.

### (d) Parameter fitting methodology

Where appropriate, we have used previously published parameter estimates obtained by fitting a deterministic analogue of the basic hookworm transmission model to data from the TUMIKIA trial.

The most epidemiologically important model parameters which vary spatially in the individual-based model are β, the average infectious contact with the reservoir (proportional to the basic reproduction number for direct life cycle helminths, *R*_0_), and *k*, which controls the level of heterogeneity in exposure to the reservoir. These two parameters control the endemic prevalence and intensity of infection. We assume all other parameters are shared by each pixel location. To reduce the total number of model parameters that require estimation, we assume a nonlinear relationship between $\beta_{i}$, $k_{i}\;$and the mean estimated baseline prevalence in each cell, ${\bar{P}}_{i}$,

2.1 $$\begin{array}{c} \beta_{i}=b_{0}+b_{1}{\bar{P}}_{i}^{b_{2}} \end{array}$$

and

2.2 $$\begin{array}{c} k_{i}=k_{0}+k_{1}{\bar{P}}_{i}^{k_{2}}. \end{array}$$

The model parameters used for fitting are $\left\{b_{0},b_{1},b_{2},k_{0},k_{1},k_{2},\theta \right\}$. The complex probabilistic structure of the interacting individual-based models creates difficulties for traditional likelihood-based parameter inference methods [40]. To bypass an exact calculation of the likelihood, we employ an approximate Bayesian computation (ABC) to perform parameter inference. The method works by drawing parameters from prior distributions, running the model for 100 years and accepting/rejecting parameter sets which produce summary statistics that lie within a minimum-tolerated distance of the data. This produces an approximate posterior distribution of the model parameters. We employed a sequential algorithm with decreasing tolerances, implemented in the R package easyABC, and used the sum of square deviations of prevalence across pixels as our distance metric, to produce 1000 samples from the posterior distribution [41].

## 3. Results

**Figure 1.**
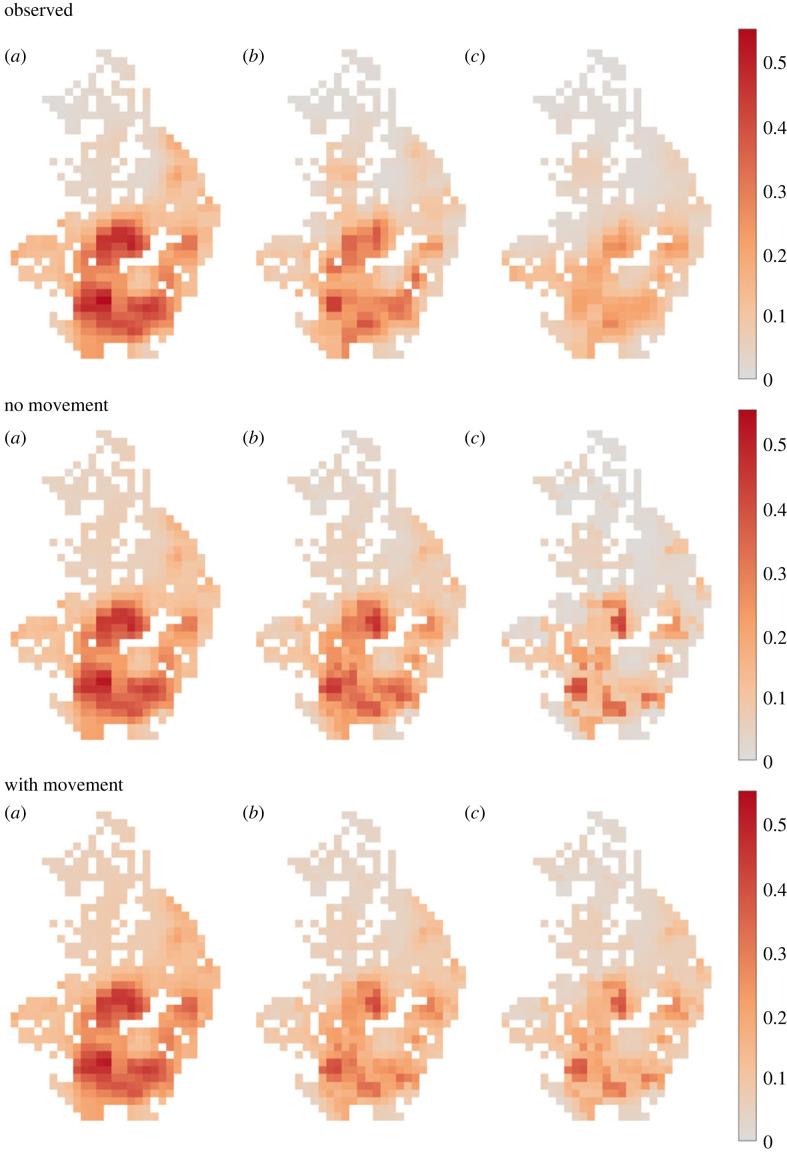
Inferred prevalence from surveys of the TUMIKIA project in Kwale province, Kenya (top row), median prevalence of no-movement model fit to baseline data, (middle row), and median prevalence of movement model fit to baseline data (bottom row) at (*a*) baseline, (*b*) midline (12 months), and (*c*) endline (24 months).

**Figure 2.**
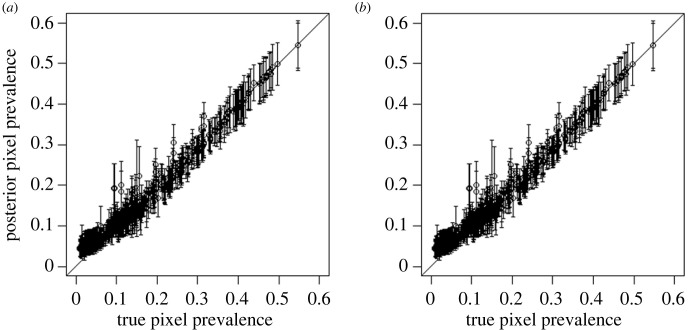
Posterior median prevalence in each pixel at baseline, plotted against true pixel prevalence, with error bars showing the standard deviation, for the model with movements (*a*) and the model without movements (*b*). The black reference line *y* = *x* represents a perfect fit to the data.

To assess the effect that human movement has on spatial transmission, we fitted our model to the baseline prevalence, with and without human movements. Panel (*a*) in figure 1 middle and bottom row, shows the median of 500 samples from the posterior distributions of the ABC model fits. The models with and without movements are able to fit well to the majority of the collected data for each site (Pearson's *r* = 0.99 in both models), however, both models have a slightly worse fit to pixels with lower than average prevalence levels (figure 2). This is possibly the result of model misspecification in equations (2.1) and (2.2), and it is feasible a better fit could be attained by using an alternative model with more parameters, although this may come at the expense of computation time and the potential for overfitting by using too many parameters. In the model with human movement, the posterior mean of the spatially averaged intrinsic *R*_0_ across pixels (not including the spatial component of transmission) was 2.63, while in the model without movement it was 2.83.

To assess the short-term accuracy of the movement model, we ran our model forward from the baseline, using 500 parameter sets drawn from the ABC posterior, and validated against the 12 months and 24 months TUMIKIA trial survey data.

In figure 1 (middle row), we observe that in the no-movement model, the different arms of the MDA programme produce large gradients of prevalence during treatment, which persist because there is no spatial transmission. By contrast, in figure 1 (bottom row), we find that human mobility is able to smooth the gradients through local movements, and reduce the effectiveness of treatment in areas which responded well to community-wide treatment in the no-movement model. The inclusion of movement produces spatial patterns of prevalence that are qualitatively much closer to the observed patterns following MDA, and both models perform reasonably well at recovering the reduction in prevalence in each arm. However, both underestimate the reduction in prevalence in the control school-aged children-only arm for reasons that are unclear at present (table 3 for mean pixel prevalence in each of the arms; table 4).

**Table 3.** Parameters and sources.

parameter	description	value	source
α	average eggs produced per female worm, per 50 mg sample of faeces (in the absence of density dependence)	3.06	[15]
β	average contact with reservoir	fitted	
γ	anti-fecundity parameter	0.01	[15]
$\delta_{w}$	average adult worm lifespan	2.0 years	[15]
$\delta_{e}$	average larval lifespan in environment	12 days	[15]
$\tau_{a}$	average human age	25 years	
Δt	timestep	1 month	
ε	proportion of adult worms killed by 400 mg dose of albenzadole	0.94	[42]
$k_{e}$	aggregation parameter of negative binomial distribution of eggs produced per female worm, per 50 mg sample of faeces	0.8	[15]
k	shape parameter of *γ* distributed predisposition	fitted	
τ	gravity model—population exponent	1.9	[35]
ρ	gravity model—distance normalization	4.3 km	[35]
σ	gravity model—kernel exponent	1.22	[35]
θ	gravity model—normalization multiplied by relative chance of exposure	fitted

**Table 4.** Mean baseline, midline and endline pixel prevalence in survey data (with 95% confidence intervals), and models with and without movements.

	arm A			arm B			arm C		
	baseline	midline	endline	baseline	midline	endline	baseline	midline	endline
data	16.5% (15.4–17.6)	11.7% (10.9–12.5)	8.0% (7.4–8.6)	17.1% (16.0–18.1)	10.0% (9.2–10.8)	6.7% (6.3–7.1)	16.5% (15.4–17.6)	13.2% (12.3–14.1)	10.0% (9.4–10.6)
no movement	17.1%	10.5%	7.6%	17.6%	10.5%	7.3%	17.1%	15.0%	13.9%
movement	17.5%	10.8%	7.9%	17.7%	10.9%	7.7%	17.4%	15.2%	14.0%

### (a) Long-term predictions following intensive mass drug administration

**Figure 3.**
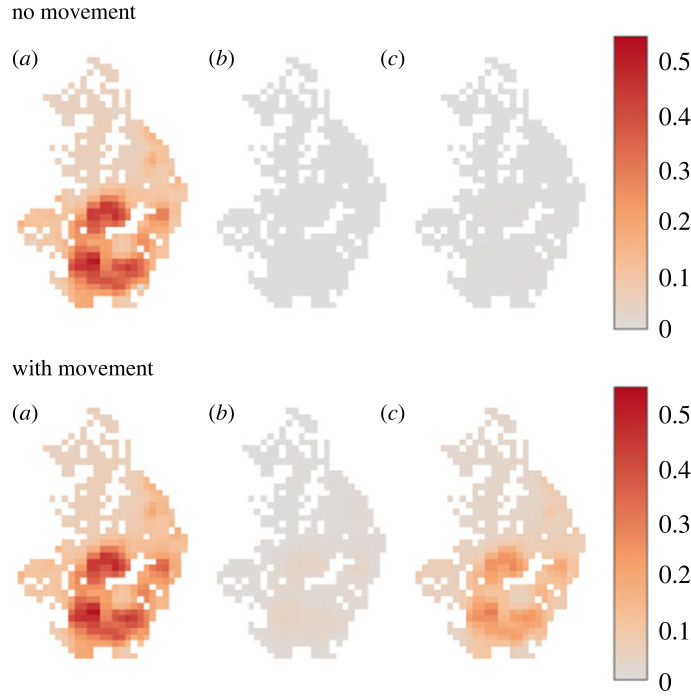
Mean prevalence of no-movement model (top row) and model with movement (bottom row) fitted to baseline data at (*a*) baseline, (*b*) after 8 years of biannual MDA, and (*c*) 10 years post MDA cessation.

To determine the possible long-term effects of human movement, we simulated a hypothetical MDA scenario, using the TUMIKIA baseline, where each pixel receives 8 years of biannual community treatment (16 rounds) with 85% coverage, and measured the prevelance after a further 10 years to assess the likelihood of infection bounce back to pre-intervention levels. In figure 3, using the no-movement model, we find that the intensive MDA treatment is able to eliminate transmission and this is maintained 10 years after MDA cessation. Similarly, the movement model is able to effectively eliminate the prevalence after MDA. However, in contrast to the no-movement model, 10 years after MDA cessation we find widespread resurgence, which as expected follows the spatial pattern of the baseline prevalence survey.

## 4. Discussion

In this study, we have developed the first explicitly spatial model of soil-transmitted helminth transmission, and applied it to hookworm infection using survey data from the TUMIKIA trial. Our model, with and without human mobility, fits well to the baseline data, reproducing the spatial prevalence pattern, although it is less accurate at recovering the prevalence within areas of low intensity infection. In future studies, a better fit may be possible by adapting the nonlinear function in equation (2.2) for *k*. This may come at the cost of computational efficiency if more parameters are required.

The spatial model is able to reproduce the pattern of the decline in prevalence as MDA is administered, although our model underestimates the overall reduction in prevalence after the fourth round of MDA. It may be possible to achieve a higher accuracy, by including the first round of MDA in the fitting methodology, or by including other parameters into the fitting methodology, such as γ, which controls the strength of the density-dependent anti-fecundity, controlling the size of individual's worm burdens. Furthermore, an assumption in our model is that prior to starting MDA treatment, the dynamics are statistically stationary. However, Kwale province had been receiving prior treatment for lymphatic filariasis, which was not accounted for in our starting assumptions at baseline.

The most important public health insight generated by our spatially explicit transmission model is that widespread bounce-back of infection is possible after a hypothetical intensive MDA scenario. In the absence of movement between villages, the intensive MDA would have eliminated transmission through the region. This result is important, since all locations in the simulated scenario reached very low prevalence levels, hence it would appear unlikely that a very small number of infected people are able to cause resurgence in their pixel and also take trips to repeatedly seed external pixels. We believe the observed bounce-back occurs because the movement model creates a small amount of positive feedback, and the large number of individuals simulated makes relatively rare events on an individual level, highly probable when summed over the entire population.

The spatial model contains many assumptions and caution should be exercised over the generalization of the results. More simulations are required to explore how different movement patterns and scales for each pixel (3 km × 3 km in the present calculations) influence the outcomes of differing MDA programmes in terms of coverage, frequency of delivery and compliance patterns. For example, consider two particular issues. First, the predictions rely on the gravity model based on data captured via mobile phones from Namibia chosen to represent human movement in Kwale, Kenya. This cannot capture precisely the real movement patterns of the population in Kwale sub-county so caution should therefore be applied before placing too great a weight on these findings. Also, it is not clear to what extent these results are generalizable to other regions. More rural, or urban, social geographies will change the volume and nature of movements between villages, and level of endemicity of the helminth parasite (underlying *R*_0_ values at any given spatial site), which could have large impact on the predictions. Other scenarios and datasets will be explored in ongoing simulations. The model described, however, provides a template on which to build an understanding given better data on movement patterns.

Second, in this study we have used a spatial grid resolution of 3 km × 3 km, with the assumption that each human host in the pixel (i.e. mirroring a village) is in contact with the same infectious reservoir. Hookworm infectivity can be very focal owing to both environmental conditions and behavioural habits, and it is possible that this scale assumption is poor in low transmission settings. This can be explored using the described model framework by using higher grid resolutions, although careful consideration is needed about whether movement patterns at a fine spatial scale be accurately captured, as the gravity model used in this study may not be appropriate on all scales. These are topics for future exploration. We also assume in this study that the majority of an individual's trips of duration of less than a day, for example, commuting to work or travelling to school, occur within the individual's 3 km × 3 km pixel. This may be a weak assumption that underestimates the magnitude of spatial transmission, and therefore data, or more realistic models, describing travelling habits of specific populations will improve the model's accuracy [43].

In conclusion, we have demonstrated in this paper the potential role that human movement has on sustaining helminth parasite transmission across linked communities. This in turn is important for the design of public health interventions and associated monitoring and evaluation programmes with respect to deciding what is a sensible spatial scale over which to administer control impact and measure outcomes. These results are pessimistic in the sense that MDA coverage levels that are predicted to permit transmission elimination without extensive people movements are invalidated when much movement occurs between communities. They bring into question the long-term effectiveness of mass deworming as a standalone method of control, and suggest that lasting elimination will require MDA interventions to be accompanied by large-scale improvements to sanitation and hygiene. These must also occur on a large spatial scale such that the effective reproductive numbers for the parasite at each spatial location are reduced to below unity in value.

## Data Availability

A version of the code used to generate results can be found from the GitHub repository: https://github.com/bcollyer/ParaSim. All data collected in Kwale sub-county, Kenya is owned by Kenya Medical Research Institute.
